# Confocal Endomicroscopy of Colorectal Polyps

**DOI:** 10.1155/2012/545679

**Published:** 2012-01-26

**Authors:** Vivian M. Ussui, Michael B. Wallace

**Affiliations:** Department of Gastroenterology, Mayo Clinic, 4500 San Pablo Road, Jacksonville, FL 32224, USA

## Abstract

Confocal laser endomicroscopy (CLE) is one of several novel methods that provide real-time, high-resolution imaging at a micron scale via endoscopes. CLE has the potential to be a disruptive technology in that it can change the current algorithms that depend on biopsy to perform surveillance of high-risk conditions. Furthermore, it allows on-table decision making that has the potential to guide therapy in real time and reduce the need for repeated procedures. CLE and related technologies are often termed “virtual biopsy” as they simulate the images seen in traditional histology. However, the imaging of living tissue allows more than just pragmatic convenience; it also allows imaging of living tissue such as active capillary circulation, cellular death, and vascular and endothelial translocation, thus extending beyond what is capable in traditional biopsy. Immediate potential applications of CLE are to guide biopsy sampling in Barrett's esophagus and inflammatory bowel disease surveillance, evaluation of colorectal polyps, and intraductal imaging of the pancreas and bile duct. Data on these applications is rapidly emerging, and more is needed to clearly demonstrate the optimal applications of CLE. In this paper, we will focus on the role of CLE as applied to colorectal polyps detected during colonoscopy.

## 1. Purpose of Paper

The purpose of the paper is to assess the importance of probe-based confocal endomicroscopy on colorectal polyps with a particular emphasis on distinguishing hyperplastic from neoplastic polyps.

## 2. Recent Findings

New endoscopic imaging modalities have emerged within the past few years and have created a new concept towards diagnosis. Recently, we have seen the evolution of endoscopy from high-definition white light endoscopy and color-enhancement methods, to the novel electronic optical technology known as “virtual biopsy,” with increased magnification now approaching that of light microscopy. Color-enhancement methods include chromoendoscopy with topical dyes, narrow-band imaging (NBI), autofluorescence endoscopy, Raman spectroscopy probes, and trimodal spectroscopy [1].

Also, Fujinon intelligent color enhancement (FICE) and iScan (Pentax) are examples of computed virtual chromoendoscopy imaging, both different from NBI, which do not depend on optical filters [2].

Confocal laser endomicroscopy (CLE) allows real-time imaging of the GI tract at approximately 1000-fold magnification with resolution of approximately 1 micron. At this level, visualization of mucosa and lamina propria, as well as single cells is achievable with real-time acquisition speeds [3, 4].

A standard classification system has been developed for both eCLE, termed the Mainz classification [3], and for pCLE, termed the “Miami” classification [5]. These systems distinguished neoplastic from hyperplastic polyps of the colon based on a dark, irregularly thickened epithelial layer characteristic of epithelial dysplasia.

## 3. Introduction

Colorectal cancer (CRC) is the fourth most commonly diagnosed cancer among men and women and ranks as the second most common cause of death from cancer in the United States [6, 7].

The importance of colonoscopy with polypectomy to reduce colorectal mortality is well known [8].

The primary purpose of colonoscopy is to identify precancerous polyps and remove them before they become malignant. Extensive studies have identified adenomatous polyps (and some serrated polyps) as the primary precursor of colorectal cancer. Other polyps such as small hyperplastic polyps especially those in the distal colon have little malignant potential. A fundamental limitation of colonoscopy is that, until recently, the only method to reliably diagnose adenomatous and hyperplastic polyp was to remove them and examine them histologically. This reliance on histology implies that there is an undesirable cost and a small risk of removing polyps with little neoplastic risk.

Hyperplastic polyps represent around one-third to one-half of all the polyps [9, 10]. Also of importance are the serrated adenomas. These types of adenomas are manifested in two varieties: traditional serrated adenoma typically assuming a polypoid appearance and the sessile serrated adenoma, flat or slightly raised and located on the proximal colon. This type of adenoma presents some molecular features different from the other colon adenomas and might develop a malignant presentation through a so-called “serrated neoplasia pathway [11].”

Although the absolute risk of polypectomy is small, it is still the most important cause of complication of colonoscopy [12]. Because of the increasing ability to distinguish hyperplastic and neoplastic polyps in vivo, as well as the increasing cost of pathologic examination of all polyps, the concept of using in vivo diagnosis to direct polypectomy has emerged. There are two potential applications of this: first, direct polypectomy only to neoplastic (and serrated) polyps and leave small, low-risk hyperplastic polyps in situ. The second application, termed “diagnose and discard,” relies on in vivo diagnosis of low-risk adenomas followed by resection without further histology.

Considering the information above about colon polyp characteristics with one-third to one-half of these polyps being hyperplastic, smaller than 10 mm, and with very low likelihood of malignancy potential [10], selecting the right polyps to remove is of utmost importance. This has the potential to reduce the risk of complications, eliminate unnecessary costs for histopathology analysis, and save time when making a decision for the patient's treatment. Several groups, including the American Society for Gastrointestinal Endoscopy (ASGE), have provided guidelines for when it would be clinically acceptable to adopt a virtual biopsy approach. They set clear thresholds for the accuracy that must be achieved (at least 90% negative predictive value for adenomatous polyps and at least 90% accuracy for predicting the correct surveillance interval). They also set clear target lesions (small distal polyps) that are both the most common polyps as well as those at lowest risk of malignant degeneration in the unlikely event that an incorrect diagnosis is made [13].

Many advanced endoscopic imaging techniques, including dye-based chromoendoscopy and digital chromoendoscopy, have been carefully studied with these goals in mind.

Some impractical aspects of dye-based chromoendoscopy, such as longer procedure times, different dyestainings, and washing techniques, contributed to its limited application. Although these factors do not affect the “virtual” chromoendoscopy methods, such as narrow-band imaging (NBI) or Fujinon intelligent colon enhancement (FICE), an extensive review by the ASGE on these methods shows modest and variable accuracy [13]. CLE has also been extensively studied with regard to colorectal polyps [3, 4].

## 4. Confocal Systems/Methods

There are currently two clinically available CLE systems; one that is integrated into a Pentax endoscope (eCLE) with the confocal imaging window located at the distal tip of the endoscope. This system allows resolution of approximately 0.8 microns, as well as variable depth of focus from the surface of the lens (0 microns) to a depth of 250 microns by using a control built into the endoscope handle. Still images are obtained at approximately 1 per second.

The second system is based on a through-the-scope probe (pCLE), produced by Mauna Kea Technologies. With pCLE, the laser scanning unit is mounted outside the endoscope, and a bundle of optical fiber, approximately 2.5 mm in diameter, delivers light and collects the images. Proprietary algorithms correct for image distortion of the long fiber bundle. The pCLE system has slightly less resolution (1 micron) and a fixed imaging depth (50 microns); but allows for faster video-rate scanning (12 frames per second) and a far greater versatility with any endoscopic system, including cholangioscopy, through the needle probes for intratumor/intracystic imaging and other non-GI endoscopes (cystoscopy, bronchoscopy, etc.).

### 4.1. Clinical Image Acquisition

All CLE imaging systems are optimized by a contrast agent, such as fluorescein sodium, discussed in detail below. The quality of the image is affected by several technical factors such as the timing of injection, probe position, and probe stability.

The optimal timing of imaging is obtained within the first 8–10 minutes after contrast injection, although acceptable imaging can be obtained for up to 60 minutes after injection [14]. In order to minimize tissue and vascular leak artifact, the probe should be placed gently in direct contact with the tissue without pressure or trauma.

The probe should be as perpendicular to the mucosa as possible. This can be challenging in the esophagus. Maintaining probe stability is critical to good image acquisition. With the eCLE system, suction is applied to the tissue immediately adjacent to the target lesion to hold the image steady. With pCLE, using the free-hand method can be challenging, but is facilitated by use of a clear 4 mm cap on the tip of the endoscope with slight suction, which can help keep the probe in the proper site.

Previously published studies with eCLE have used topical acriflavine dye, but its use has been diminished as a consequence of the possibility of damage to the DNA cells. Alternately, fluorescein, a relatively inexpensive and safe contrast agent already approved by the FDA for diagnostic angiography and angioscopy of the retina, has been commonly used during pCLE procedures. It can be used topically and intravenously with an excellent safety profile [15].

One to five mL intravenous injection of a 10% solution enables the visualization of each cell, intensely contrasting the capillary network [14, 16, 17]. The initial images in the first 10–30 seconds are predominantly vascular; however as fluorescein leaks into the extravascular space, epithelium and stromal tissues are visualized. Neoplasia tissue appears as dark epithelial cells, which could be explained by the lack of fluorescein absorption, accelerated expulsion from the cell, or greater leakage into the lamina propria [5].

The lack of direct nuclear visualization does not allow the comparison between nucleus and cytoplasm and, therefore, cannot be used for diagnosis and grading intraepithelial malignancies [18]. However, the contrast of the dark color of the neoplastic epithelium permits architectural analysis of the surface mucosa and aids in differentiating normal mucosa from neoplastic tissue [19].

The major side effects of fluorescein are temporary, lasting up to 24 hours and consist of skin discoloration and a yellowish tint to the urine. Other complications include nausea, hypotension, and mild skin rash. Serious complications, such as anaphylaxis or infection site reactions, are extremely rare [15].

### 4.2. Grading Confocal Images

The Mainz criteria, developed by Kiesslich et al. [3] in 2004, described patterns of normal, regenerative, and neoplastic tissue as seen by eCLE (Table 1). Sanduleanu et al. [20] further defined the key patterns in colorectal polyps (Table 2).

**Figure 1:**
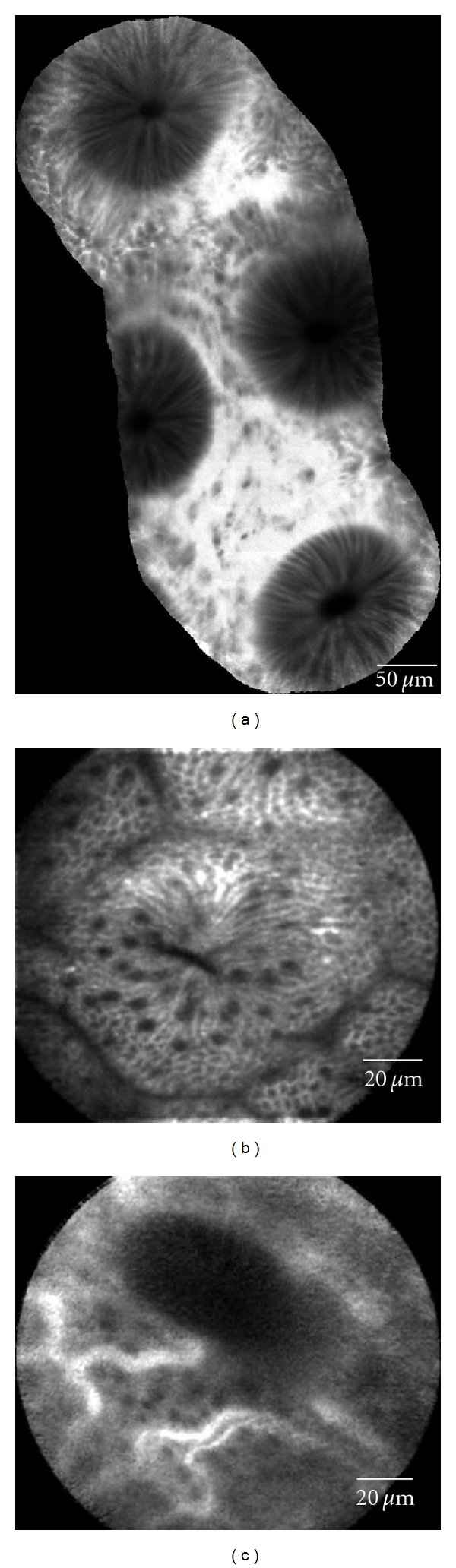
Normal colon epithelium. (a) Mosaic image of normal crypts, which are circular and evenly distributed. Although the epithelium is relatively dark compared to the bright stroma, the thickness is very regular with uniform crypt structures. (b) Single crypt opening, slightly slit-like but with abundant goblet structures (dark “dots” within the epithelial cells). (c) Capillary vasculature typically seen very early in the injection. The vessels are small (<10 mm) in diameter and form a regular capillary network.

**Figure 2:**
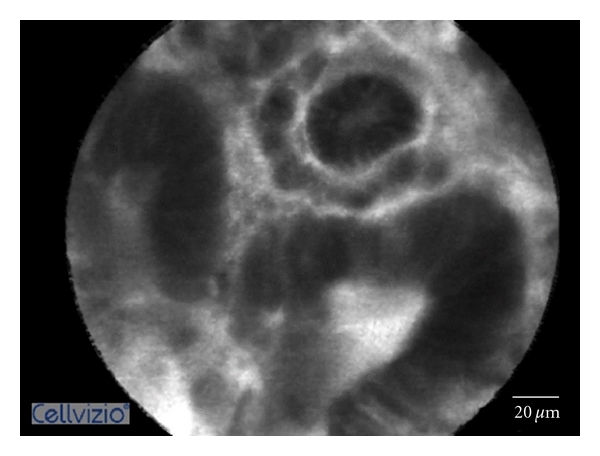
Adenomatous polyps. The epithelium is much darker with irregular thickness and forming small villous-like structures with wide openings.

**Figure 3:**
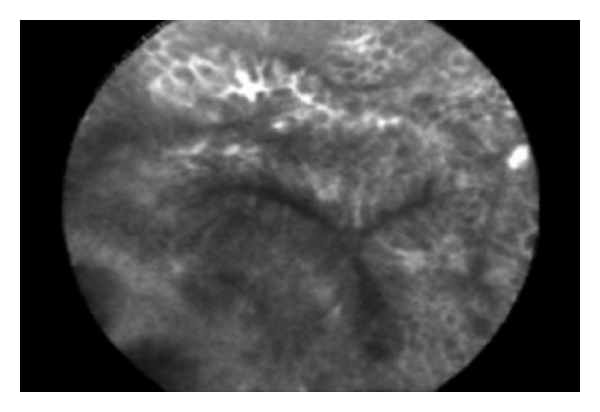
Hyperplastic polyp. The crypt opening is stellate in shape similar to Kudo type 2 pit patterns. The epithelium is relatively bright and regular in thickness.

Finally, the Miami classification system, which is similar to the Mainz system, was developed specifically for pCLE images (Table 3) [5]. Examples of normal colonic tissue are shown in Figures 1(a)–1(c). Examples of colonic adenomas and hyperplastic polyps are shown in Figures 2 and 3.

## 5. Clinical Application

### 5.1. Colon Polyps

The following methods have been applied to predict histology based on the Kudo pit pattern, vascular pattern intensity (VPI), and color: Chromoendoscopy (CE), narrow-band imaging (NBI) and a combination of NBI and autofluorescence imaging (AFI) called endoscopic trimodal imaging (ETMI), and CLE. Regarding colon polyps and CLE, the most significant reason for its application is the capability of discerning a hyperplastic from an adenomatous polyp.

The first report of the use of endoscope-based CLE was published by Kiesslich et al. [3]. Their study of 42 patients has proven the ability to predict the presence of neoplastic alterations with a very high degree of accuracy (sensitivity, 97.4%; specificity, 99.4%; accuracy, 99.2%).

De Palma et al. [21] further reported the accuracy and interobserver agreement for pCLE in colorectal polyps. In a study involving 32 small polyps in 20 patients ranging from 1 to 9 mm, comparing pCLE and NBI, pCLE achieved a sensitivity and negative predictive value of 100% and specificity of 85% in predicting adenomatous histology.

Our group recently reported a comparison of pCLE to virtual chromoendoscopy (NBI or FICE). Sensitivity of pCLE was higher than virtual chromoendoscopy (91% versus 77%) *P* = 0.01 with similar specificity (76% versus 71%). When looking at subgroups of polyps imaged NBI versus FICE, the advantage of pCLE was only seen in comparison to FICE imaging with statistically similar accuracy when compared to NBI [22]. This study included both large (>9 mm) and small polyps. Therefore, our group has further explored the accuracy of pCLE, whereas NBI focused exclusively on the small polyps that may be eligible for a diagnose-and-discard strategy. In this study, pCLE and NBI were evaluated both independently and in combination.

One hundred and thirty polyps <10 mm were evaluated in 65 patients. pCLE had a higher sensitivity than NBI (86% versus 64%, *P* 0.008) but with lower specificity (78% versus 92%, *P* 0.027) and similar overall accuracy. When combining pCLE and NBI, limiting the analysis to high-confidence images, the sensitivity and negative predictive value was 94% and specificity 97%. This is important as it demonstrates the technology can exceed the ASGE recommended thresholds (90% or greater) for acceptance of a diagnose-and-discard strategy [23].

The previous trials of both eCLE and pCLE have largely evaluated offline image interpretation, which does not allow on-table decision such as diagnose and discard. Our group has recently compared real-time and offline pCLE interpretations of colorectal lesions. Although real-time interpretation accuracy was slightly lower (78% versus 81%), these differences were statistically equivalent [23, 24].

An article assessing the learning curve of in vivo pCLE for prediction of colorectal neoplasia indicated that a wide range of GI specialists could become proficient in interpreting high-quality pCLE images after review of 50–70 cases and approximately 2 hours of training [9].

## 6. Conclusion

Confocal laser endomicroscopy has brought several insights and new concepts to the endoscopic field. Considering all the advantages and drawbacks, it is important to highlight a few aspects.

1. Though the learning curve does not appear to be long, some training is required to achieve a consistently high level of accuracy.
2. The combination of virtual chromoendoscopy, such as NBI and CLE, has proven to be a highly accurate method for in vivo diagnosis, allowing a careful virtual panchromocolonoscopy followed by target endomicroscopic examination.
3. pCLE has shown higher sensitivity but similar specificity compared to NBI for small polyps, especially those sized 1–5 mm.
4. Although promising, the cost of CLE is still high relative to histology. In order to become more clinically relevant, the cost must be reduced through more durable, less expensive probes and integrated endoscopic devices.

## Figures and Tables

**Table 1:** Colorectal pathology prediction using confocal pattern classification.

Grade	Vessel architecture	Crypt architecture
Normal	Hexagonal, honeycomb appearance	Regular luminal openings, homogenous layer of epithelial cells
Regeneration	Hexagonal, honeycomb appearance with no or mild increase in the number of capillaries	Star-shaped luminal crypt openings or focal aggregation of regular-shaped crypts with a regular or reduced amount of goblet cells
Neoplasia	Dilated and distorted vessels; irregular architecture with little or no orientation to adjunct tissue	Ridged-lined irregular epithelial layer with loss of crypts and goblet cells; irregular cell architecture with little or no mucin

**Table 2:** Systematic classification of colorectal lesions (eCLE based).

	General architecture	Cytonuclear features
Normal mucosa	Regular (uniform) architecture of surface and glandular epithelium	Epithelial cells are uniformly lined up along the basement membrane
	Regular “honey-comb” appearance of vascular pattern	Normal cell polarity of surface and glandular epithelium, normal aspect of mucin-producing goblet cells
Nonadenomatous polyps	Slightly disturbed architecture: enlarged, branch-like, elongated crypts	Epithelial cells are morphologically normal, preserved cell polarity
	Increased number of cells in the crypts	Depletion of goblet cells
	Mild alterations of vascular pattern	
	Inflammatory infiltrate of lamina propria, decreased crypt/stroma ratio	
Adenomatous polyps	Disturbed architecture: mild irregularity of the crypts, eventual villous transformation, increased crypt/stroma ratio, crypt destruction	Incomplete to lack of epithelial surface maturation
	Mild to moderate alterations of vascular pattern	Slightly cytonuclear atypia
		Islands of malignant cells

**Table 3:** Potential applications of pCLE or CLE.

Areas that have been well evaluated	Barrett's esophagus guide to biopsy
	Colon polyp classification

Areas of early exploration	Inflammatory bowel disease—dysplasia
	Biliary strictures
	Duodenal neoplasia

Experimental areas	Solid and cystic tumor imaging
	Gastric neoplasia
